# GEROPSYCHOLOGY STUDENTS’ EXPERIENCES OF THE INTERNSHIP MATCH AND IMPLICATIONS FOR TRAINING

**DOI:** 10.1093/geroni/igz038.2226

**Published:** 2019-11-08

**Authors:** Lisa Bloom-Charette

**Affiliations:** ENRM Veterans Hospital, Bedford, Massachusetts, United States

## Abstract

There has been little literature on the effectiveness of the clinical psychology internship match program within the specialty of geropsychology. This study reports student responses to a 2018 survey conducted by the Council of Professional Geropsychology Training Programs (CoPGTP). Students reported that they completed a mean of 18.4 (SD = 7.23) internship applications, and had a mean of 8.4 (SD = 2.3) interviews. Compared to generalists, geropsychology students applied to and interviewed at more programs, felt slightly less supported, but matched more frequently at their first choice site. All respondents matched to generalist rather than geropsychology programs, and all matched to their first ranked programs. The majority of students felt strongly supported, and noted that seminars, essay review, and the Pike’s Peak training model were important resources. Reasons for not choosing geropsychology programs included geographical preferences and the belief that adequate geropsychology training can be obtained in a generalist track.

